# “Looks Can Be Deceiving”: Adrenal Teratoma Causing Diagnostic Difficulty

**DOI:** 10.1155/2015/232591

**Published:** 2015-12-16

**Authors:** Mehwash Nadeem, Muhammad Hammad Ather, M. Nasir Sulaiman, Shahid Pervez

**Affiliations:** ^1^Section of Urology, Department of Surgery, Aga Khan University, P.O. Box 3500, Karachi,, Pakistan; ^2^Section of Histopathology, Department of Pathology, Aga Khan University, P.O. Box 3500, Karachi, Pakistan

## Abstract

Teratomas are unusual tumours that derived from totipotent cells with their origin from more than one or usually all three germ cells. Here authors are presenting a case of primary retroperitoneal tumour that is a rare clinical entity. A 19-year-old male presented with right lumbar pain and was found to have complex cyst with large calcification in right adrenal gland on imaging. Intraoperatively, he was found to have a solid mass with areas of soft consistency, which was excised en bloc. On gross examination, the cyst contained pieces of bone, few teeth, and hairs entangled in mucinous material. On histological evaluation, it was confirmed to be mature teratoma arising from the right adrenal gland. He made uneventful recovery and was kept well on annual follow-up.

## 1. Introduction

Teratomas are rare tumours that shares radiological features with many other benign conditions. Although definitive diagnosis is possible on histopathological evaluation only, pertinent radiological finding mentioned in this report can help others to suspect this rare yet important clinical entity.

This case highlights the importance of correct and meticulous interpretation of the radiological investigation.

## 2. Result

### 2.1. Case Presentation

A 19-year-old male with no prior health issues presented with complaint of vague pain in right flank for the past 9 months. He described the pain as intermittent, nonradiating with no specific aggravating or relieving factors. Past history was unremarkable. On examination, he appears healthy with normal blood pressure and pulse rate. Abdominal and rest of the systemic examinations did not reveal anything significant.

### 2.2. Investigations

His baseline workup including complete blood count, renal profile, and liver function tests was within normal limits. His haemoglobin was 128 g/L (normal: 121–160 g/L) and serum creatinine was 0.8 mg/dL (normal: 0.7–1.2 mg/dL). Based on the findings of imaging, investigations were ordered to exclude functional adrenal tumour although the patient was completely asymptomatic. 24-hour urinary cortisol was 89 mic gm/24 hours (normal range: 55.5–286 mic gram/24 hours) and serum cortisol (dexamethasone suppression) was 0.9 mic gram/dL (normal range: less than 2 mic gram/dL).

**Figure 1:**
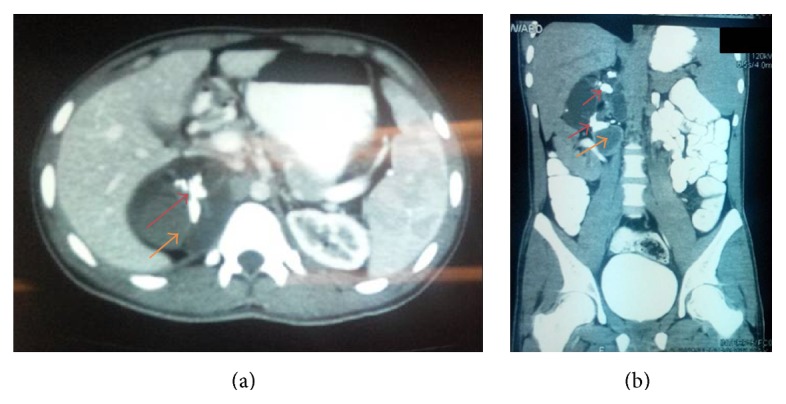
Contrast enhanced CT scan showing cystic mass with solid component (marked with orange arrow) and areas of calcification (marked with red arrow). (a) Axial section. (b) Coronal section.

Contrast enhanced computed topography (Figure 1) revealed a large cyst arising from right adrenal gland with mixed density that raised suspicion of malignant tumour. Other abdominal structures were normal and there was no evidence of distant metastasis although the right kidney was slightly displaced by the mass.

### 2.3. Differential Diagnosis

The most important differential diagnosis based on the imaging could be teratoma, angiomyolipoma, or myelolipoma that shares few radiological features and causes diagnostic difficulty. Confirmation of diagnosis in this case is only possible by histopathological evaluation of resected tissue; however, in this particular case the presence of large calcification (which was actually a piece of bone) on CT scan was an important finding that is not seen with the above-mentioned clinical entities.

### 2.4. Treatment

The patient underwent en bloc excision of the mass through flank approach with resection of 11th rib. Mass of about 8 × 6 × 4 cm was found just above the kidney having bosselations over its surface with different areas of soft and hard consistency. It was densely adhered to the surrounding structures, so careful dissection was done to define the planes. Tumour was removed and sent for histopathological evaluation.

### 2.5. Outcome and Follow-Up

**Figure 2:**
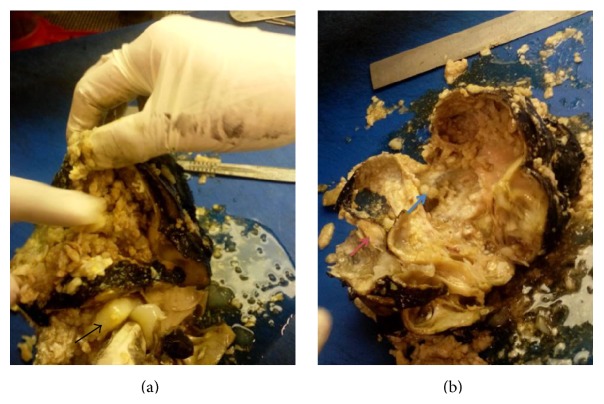
Gross examination of specimen, piece of cartilage (black arrow), bone (pink arrow), and teeth (blue arrow) with mucinous substance.

**Figure 3:**
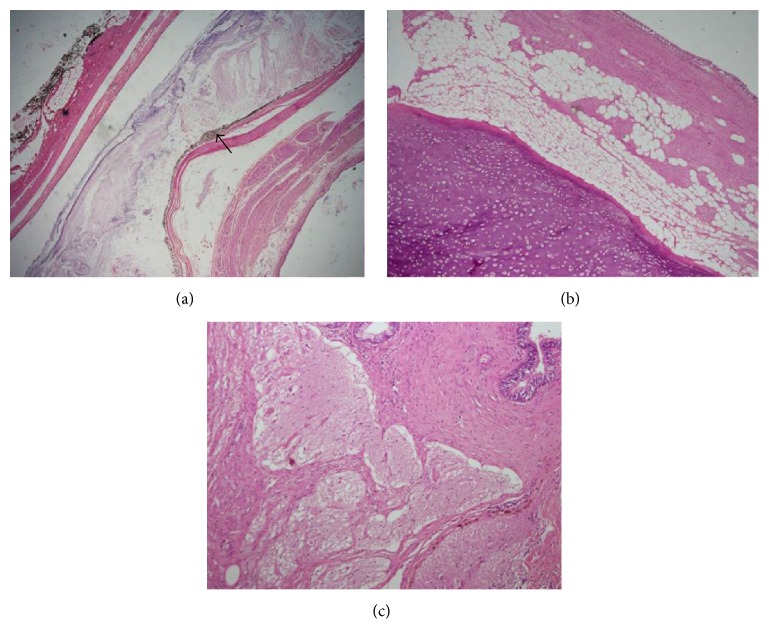
(a) Low power view showing choroid differentiation. Please note heavily pigmented melanocytes giving black coloration (arrow); H&E stain, 10x. (b) Respiratory lining with smooth muscle, adipose tissue, and mature hyaline cartilage; H&E stain, 10x. (c) Mature glial tissue with collagenous stroma. Focally mature glandular epithelium is noted. H&E, 20x.

The patient made excellent postoperative recovery and was discharged home. On gross evaluation, multiple structures including teeth, small piece of bone, a piece of cartilage, and few hairs were found within the cyst entangled in mucinous material (Figure 2). Histology of the specimen showed a widely varied differentiation representing all three germ layers. All tissues identified however were completely mature. Preferential differentiation included various types of epithelia, choroid, and glial tissue as well as cartilaginous, bony tissue, muscular, and adipose tissue (Figure 3). The adrenal tissue was largely replaced by teratoma. These findings confirmed the adrenal lesion to be benign mature cystic teratoma.

On follow-up at the 12th postoperative day, the patient was doing well and the wound was completely healed. Histopathology was discussed with the patient and he was advised about follow-up after 6 months. He was last seen after a year of surgery and was completely asymptomatic. His repeated ultrasound was unremarkable as well.

### 2.6. Discussion

Teratoma is a rare neoplasm with incidence of 0.9/100,000 population [1, 2]. Teratomas that occur in infancy and early childhood are usually extragonadal, whereas those found in older children are more commonly located in the gonads [3]. It represents an infrequent entity when found as primary retroperitoneal neoplasm in adults [4].

Since teratoma originates from pluripotent cells of two or more than two germ cell layers, the contents can be different. In mature teratoma, range of adult tissue types can be found including skin, muscle, nerve, fat, and tooth structures [5].

The majority of patients are asymptomatic while others may present with nonspecific complaints like backache or flank or generalized abdominal pain although cystic teratomas may rupture and cause sudden onset of abdominal pain, ascites, and peritonitis [5, 6]. High index of suspicion supplemented with diagnostic workup (mainly imaging) helps diagnosing this rare tumour although only histopathological examination is confirmatory. The common radiological findings of teratoma, described in the literature, are cystic masses with or without calcifications that may mimic angiomyolipoma or leiomyoma [7]. Our patient had very big calcification on CT scan with few mixed density areas unlike other cases where calcification was small or absent [8]. That calcification was later found out to be a piece of cartilage within the cyst. This distinct feature makes this case unique and we may suggest that the presence of large calcification within cystic retroperitoneal mass should be considered as distinguishing feature for teratoma from other retroperitoneal masses. Diagnosis on the basis of these key findings may help in better preoperative planning and patient counselling.

This case is one of the very few cases reported in the literature so far, latest by Sasi and coworkers [9] who report similar tumour, but in a 28-year-old female on left side causing abdominal pain and distension. Our patient shares very similar radiological and histopathological features except the age, mode of presentation and pertinent radiological features.

These tumours are likely to be benign and carry excellent prognosis after treatment with overall 5-year survival nearly 100% [10].

## 3. Conclusion

Teratoma is a rare but important differential diagnosis of retroperitoneal masses. High index of suspicion and radiological investigation are the mainstay of diagnosis. The presence of large calcification is an important finding that may help distinguishing teratoma from other masses. Histopathological evaluation is mandatory to exclude any component of malignancy.
